# A guilt-by-association mutation network in LGL leukemia

**DOI:** 10.18632/oncotarget.21699

**Published:** 2017-10-09

**Authors:** Emma I. Andersson, Alessandro Coppe, Stefania Bortoluzzi

**Affiliations:** Stefania Bortoluzzi: Department of Molecular Medicine, University of Padova, Padova, Italy

**Keywords:** large granular lymphocyte leukemia, somatic mutations, mutation network, systems genetics, WES

Large granular lymphocyte leukemia (LGLL) is a rare clonal disease characterized by an excess of CD8+ cytotoxic T cells or natural killer (NK) cells and associated with severe cytopenias, recurrent infections, and autoimmune diseases. Known LGLL hallmarks are JAK/STAT pathway activation, deregulation of pro-apoptotic pathways (sphingolipid and FAS/FAS ligand), and activation of pro-survival signaling pathways (PI3K/AKT and RAS). Aberrant STAT signaling is observed in LGLL due to somatic mutations or to other mechanisms, such as epigenetic inactivation of JAK-STAT pathway inhibitors and increased interleukin-6 secretion. Activating STAT3 somatic mutations are frequently (30-70%) present in the SH2-domain [1] and very rarely (2%) in the DNA-binding or coiled-coil domain [2]. STAT5B mutations are more rare, but typical of CD4+ T-LGLL (50%) [3].

In a recent study [4] consisting of 19 LGLL patients, we characterized the genomic landscape of somatic mutations in LGLL including STAT-mutation negative cases. Altogether, 347 different genes carrying high confidence rare somatic variants with a strong functional impact were discovered. In addition to genes recurrently mutated, novel genes emerged from functional prioritization by a system genetics approach.

Different LGLL patient subgroups, albeit showing similar clinical characteristics, had different mutation profiles with significantly higher mutation burden in CD4+ T-LGLL, possibly in relation to cytomegalovirus-derived stimulation and restricted usage of TCR Vβ.

In addition to mutations of STAT3 in CD8+, and of STAT5B both in CD4+ and in CD8+ cases, 14 genes were recurrently mutated, including transcriptional/epigenetic regulator, tumor suppressor and cell proliferation genes. Of them, both KMT2D histone methyltransferase, and PCLO calcium sensor regulating cAMP-induced exocytosis were previously linked to lymphomagenesis. Two genes, ARL13B and FAT4, recurrently mutated in a mutually exclusive way were involved in control of cell growth by Hippo signaling, and additional non-recurrent somatic mutations in YAP1 and in its inhibitor AMOTL1 pointed towards an involvement of Hippo pathway deregulation in LGLL. In addition to STAT5B, also HRNR, encoding a calcium-binding protein involved in hematopoietic cell differentiation, had recurrent mutations in CD4+ T-LGLL. The three STAT mutation negative NK-LGLL cases harbored somatic mutations in 31 genes including several “cancer genes” as KRAS, PTK2, NOTCH2, CDC25B, HRASLS, RAB12, PTPRT, and LRBA, none of them shared by the subjects.

**Figure 1:**
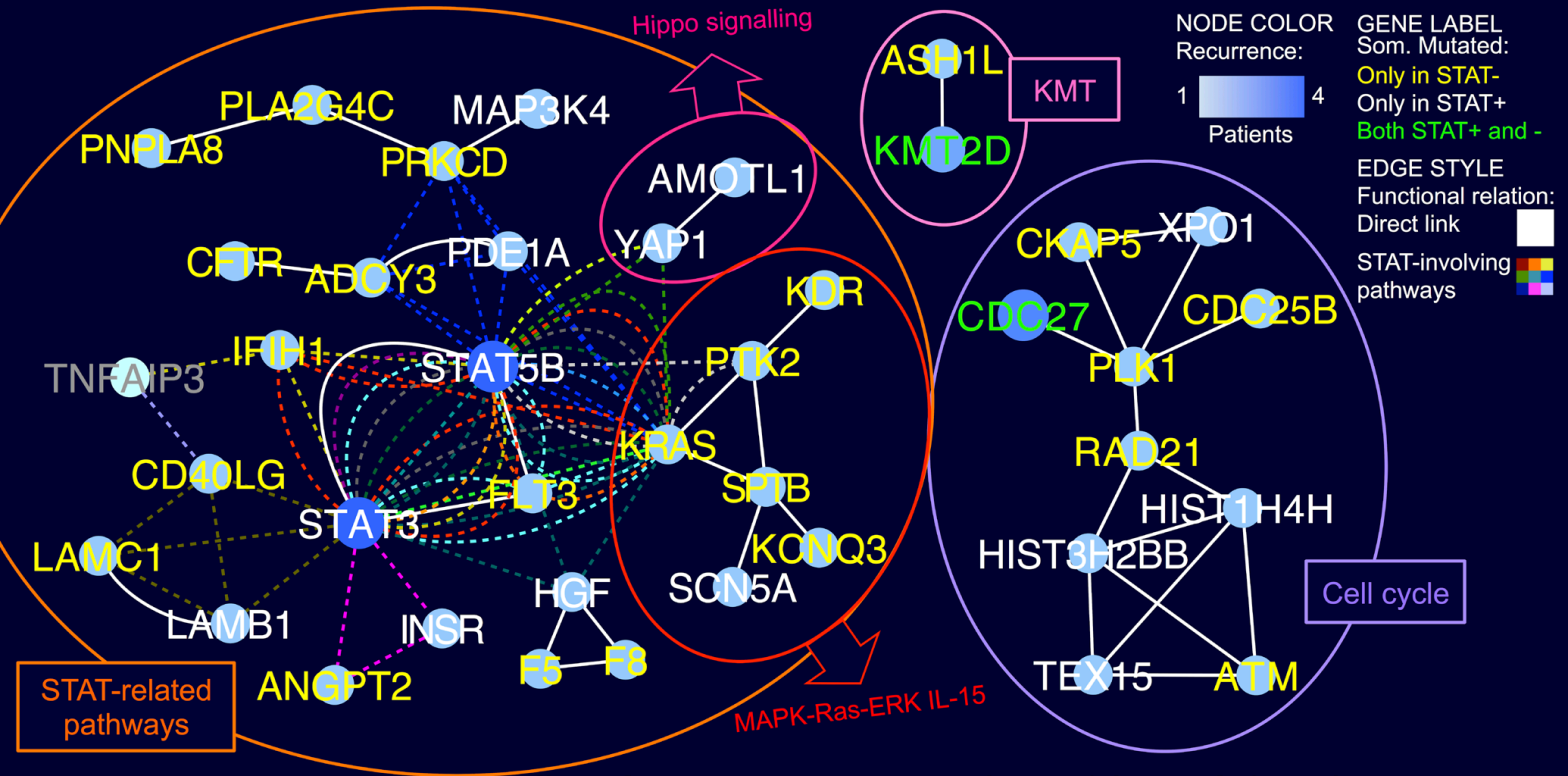
Core LGL leukemia mutation network The network shows the functional relations of genes somatically mutated in LGLL patients, according to the integration of KEGG and Reactome pathway topologies. Network nodes represent mutated genes, with the node and label colors indicating respectively the recurrence in the cohort, and if the gene was mutated only in STAT-mutation-positive (STAT+), only in STAT-mutation-negative (STAT−), or in both patient groups, as shown in the legend (TNFAIP3 is shown in grey as not mutated in the cohort). White solid lines indicate direct interactions and colored dashed lines connect genes participating in pathways including STAT3 and/or STAT5B.

Since mutations hitting different functionally connected genes can drive a similar phenotype and concur to it if present in the same patient, we next implemented a knowledge-based systems genetic approach reminiscent of that developed for expression data-driven network modeling of cancer cells [5,6]. The prioritization of genes and functions emerged by a “guilt-by-association” analysis of the integrated pathway-derived LGLL mutation network. Mutated genes were indeed mapped to 119 KEGG and 426 Reactome pathway-derived networks, next merged in a network depicting direct interactions and functional relations between 61 genes mutated in LGLL. Albeit being prevalently mutated only in one sample each, these genes clustered in a few highly connected components indicating functional modules and sub-pathways hit by somatic mutations. The largest network component included 24 mutated genes directly linked to STAT3/5B, to their neighbors and/or participating to pathways including STAT genes (Figure 1). At least one gene of this group was mutated in all but three patients of the cohort and, remarkably, in two thirds of STAT-mutation negative tumours. On top, we detected mutation of FLT3 receptor tyrosine kinase, promoting the phosphorylation of various targets in the PI3K/AKT/mTOR, RAS, and JAK/STAT signaling pathways. Another receptor mutated in STAT-negative patients was CD40LG, which modulates B-cell and immune system function participating in STAT3 and NFAT signaling pathways, and it is also linked to MAPK-Ras-ERK and IL-15 pathways, both deregulated in LGLL. In addition, CD40LG is functionally linked to TNFAIP3, a tumor suppressor negatively regulating NF-κB signaling recently found mutated in T-LGLL [7]. KRAS, and the connected kinases PTK2 and KDR expressed in lymphocytes carried deleterious mutations in STAT-negative patients. Strikingly, several STAT-negative patients carried mutations in more than one gene of the STAT-related component and the coexistence of multiple mutations (e.g. CD40LG, F8, PLA2G4C in a CD4+, and FLT3, KDR, KCNQ3 in a CD8+) in the main LGLL clone was supported by similar variant allele frequencies.

In addition to STAT3/5B network, other two smaller network components of high interest were detected. The first comprised nine genes linked to cell cycle regulation, including CDC25B, a phosphatases controlling mitotic progression, and ATM, involved in apoptosis and p53 signaling, and the second was an epigenetic module made by ASH1L and the recurrently mutated KMT2D, both members of the multifunctional transcriptional coactivator ASCOM complex, acting also on p53.

The systems genetic approach set up in the study succeeded to map mutations found in LGLL patients into novel functional modules, reinforcing the central role of JAK-STAT network, and provided important new insights of the activation of this pathway in those LGLL cases without STAT mutations. We expect that this kind of big data analysis approaches, integrating knowledge and experimental data in mutation networks will gain importance to interpret genome sequencing results. With progressively large and complex networks, the adoption of informative gene and edges weighting, coupled with topological analyses of significant mutation clustering, will gain discovery power, generate new hypotheses and disclose relevant biological information.
